# Regenerative Medicine Approaches to Craniofacial and Corneal Neuropathic Pain

**DOI:** 10.3390/ph19050692

**Published:** 2026-04-28

**Authors:** Franzes Anne Z. Liongson, Jin Yoo, Benjamin Swett, Steven M. Falowski, Jason E. Pope, Dawood Sayed, Timothy E. Deer, Jamal J. Hasoon, David A. Keith, Fernando P. S. Guastaldi, Ronald J. Kulich, Christopher L. Robinson

**Affiliations:** 1 Department of Anesthesiology, Perioperative Care, and Pain Medicine, NYU Grossman School of Medicine, New York, NY 10016, USA; jin.yoo@nyulangone.org (J.Y.); benjamin.swett@nyulangone.org (B.S.); 2 Neurosurgical Associates of Lancaster, Lancaster, PA 17601, USA; 3 Evolve Restorative Center, Santa Rosa, CA 95403, USA; 4 Department of Anesthesiology, The University of Kansas School of Medicine, Kansas City, KS 66160, USA; 5 Spine and Nerve Center of the Virginias, West Virginia University Health Sciences, Morgantown, WV 26506, USA; 6 Department of Anesthesia, Critical Care, and Pain Medicine, UTHealth, McGovern Medical School, Houston, TX 77030, USA; 7 Division of Oral and Maxillofacial Surgery, Department of Oral and Maxillofacial Surgery, Massachusetts General Hospital, Harvard School of Dental Medicine, Boston, MA 02115, USA; 8 Department of Anesthesia, Critical Care, and Pain Medicine, Massachusetts General Hospital, Boston, MA 02134, USA; 9 Department of Psychiatry, Massachusetts General Hospital, Boston, MA 02134, USA; 10 Division of Pain, Anesthesiology and Critical Care Medicine, The Johns Hopkins University School of Medicine, Baltimore, MD 21205, USA

**Keywords:** craniofacial neuropathic pain, corneal neuropathic pain, regenerative medicine, mesenchymal stem cells

## Abstract

Craniofacial and corneal neuropathic pain are disabling conditions characterized by persistent pain that is frequently refractory to conventional pharmacologic and interventional therapies. These disorders arise from complex interactions between peripheral nerve injury, neuroinflammation, and maladaptive central sensitization within trigeminal pathways, features that span neuropathic and nociplastic pain mechanisms as defined by the International Association for the Study of Pain, thus emphasizing the need for mechanism-based, patient-stratified treatment strategies. Regenerative medicine offers a paradigm shift from symptom suppression toward structural nerve repair and functional restoration. This narrative review examines the pathophysiological mechanisms underlying craniofacial and corneal neuropathic pain and critically evaluates emerging regenerative therapies, including autologous biologics (autologous serum tears and platelet-rich plasma), mesenchymal stem cells and their derivatives, exosomes and extracellular vesicles, and neurotrophic peptides. Particular emphasis is placed on corneal neuropathic pain as a translational model, given the cornea’s dense sensory innervation and the ability to non-invasively quantify nerve regeneration using in vivo confocal microscopy as an objective biomarker of treatment response. Clinical evidence across regenerative modalities varies by indication: cenegermin has demonstrated robust efficacy and regulatory approval for neurotrophic keratitis, while platelet-rich plasma shows growing evidence in temporomandibular disorders, myofascial pain, and occipital neuralgia. Cell-based and cell-free therapies demonstrate strong preclinical promise but remain limited by heterogeneous protocols and a paucity of large-scale randomized trials. Key barriers to translation include regulatory uncertainty, lack of standardized outcome measures, and workforce and implementation challenges. Advancing regenerative therapies for craniofacial and corneal neuropathic pain will require rigorous clinical trials, biomarker-driven patient selection, and multidisciplinary collaboration. Sex as a biological variable remains underexplored across all regenerative modalities and represents a priority for future research.

## 1. Introduction

Craniofacial and corneal neuropathic pain are disabling conditions that frequently are refractory to conventional therapeutic interventions, substantially reducing patient quality of life. Corneal neuropathic pain arises from damage to corneal sensory nerves, presenting with burning, stabbing, foreign-body sensations, and photophobia [1]. The cornea’s dense sensory innervation—which protects the tear film and maintains visual function—renders it uniquely vulnerable to neuropathic transformation following injury [2]. Similarly, craniofacial neuropathic pain encompasses conditions such as trigeminal neuralgia, post-traumatic facial pain, temporomandibular disorders with neuropathic features, and occipital neuralgia, all of which involve dysfunction of trigeminal or cervical sensory pathways [3,4].

Regenerative medicine offers a shift from symptom management to tissue repair, targeting the root causes of neuropathic pain: damaged sensory nerves, dysregulated immune responses, and maladaptive central processing. Regenerative strategies—including autologous biologics (platelet-rich plasma (PRP) and serum tears), mesenchymal stem cells (MSCs) and derivatives, extracellular vesicles, and neurotrophic peptides—aim to restore normal nerve structure and function, thereby alleviating chronic pain at its source.

For clarity, PRP (platelet-rich plasma) refers to any autologous high-platelet plasma concentrate; PRGF (plasma rich in growth factors) is a specific leukocyte-free, calcium-activated PRP subtype per Anitua’s protocol; and orthobiologics is the umbrella term encompassing PRP, bone marrow aspirate concentrate (BMAC), mesenchymal stromal cells, and scaffolds.

The cornea functions as an ideal translational model. Its exceptionally dense innervation enables real-time, non-invasive tracking of nerve regeneration via in vivo confocal microscopy (IVCM), providing objective biomarkers for evaluating therapeutic efficacy [5,6]. Insights gained from corneal nerve biology—optimal neurotrophic factor dosing, immunomodulation strategies, and biomarker-driven therapy selection—can inform regenerative approaches for broader craniofacial neuropathic pain, accelerating evidence-based practice. This narrative review examines the pathophysiological basis of craniofacial and corneal neuropathic pain and evaluates regenerative therapies, highlighting evidence gaps and regulatory considerations.

### Methodology

This narrative review was prepared in accordance with the Scale for the Assessment of Narrative Review Articles (SANRA). Electronic databases searched were PubMed, Embase, Cochrane CENTRAL, and Web of Science, with a date window of January 1995 to February 2026 and restriction to English-language sources. Search-term combinations included “corneal neuropathic pain,” “trigeminal neuralgia,” “temporomandibular disorder,” “occipital neuralgia,” “platelet-rich plasma,” “mesenchymal stem cells,” “extracellular vesicles,” “nerve growth factor,” and “cenegermin.” Inclusion criteria comprised original preclinical and clinical studies of regenerative therapies for craniofacial or corneal neuropathic pain syndromes; exclusion criteria were non–peer-reviewed reports, conference abstracts without subsequent publication, and publications in languages other than English.

## 2. Mechanisms Underlying Craniofacial and Corneal Neuropathic Pain

The contemporary classification of chronic pain by the International Association for the Study of Pain (IASP) distinguishes three mechanistic categories: nociceptive pain, arising from activation of nociceptors in response to actual or threatened tissue damage; neuropathic pain, caused by a lesion or disease of the somatosensory nervous system; and nociplastic pain, arising from altered nociception despite no clear evidence of tissue damage or somatosensory nerve injury sufficient to explain the pain [7]. While craniofacial and corneal pain conditions are conventionally labeled “neuropathic,” reflecting their origin in peripheral nerve injury, their full clinical phenotype frequently extends beyond this category. As detailed in the sections below, persistent central sensitization, amplified descending facilitation, and affective-cognitive modulation are prominent features of these conditions and substantially overlap with nociplastic mechanisms. Readers should therefore understand that these disorders often represent a mixed or transitional phenotype, with neuropathic features predominating early and nociplastic features becoming increasingly prominent as chronification occurs. This mechanistic heterogeneity has direct implications for patient stratification and regenerative therapy selection, as treatments targeting peripheral nerve repair may be less effective in patients whose pain is driven predominantly by central mechanisms.

### 2.1. Peripheral Nerve Changes

In craniofacial and corneal neuropathic pain, physical insults—surgical trauma, nerve injury, infection, or inflammation—sensitize sensory afferents through cytokine-mediated alterations of nociceptor membrane properties in susceptible individuals [8]. Patients carrying certain genetic polymorphisms in nociceptor ion channels (e.g., SCN9A, TRPV1, and TRPA1) demonstrate vulnerability to the effects of inflammatory mediators. Exposure to IL-1β, TNF-α, and nerve growth factor (NGF) following injury decreases threshold potentials and increases nerve depolarization in corneal and craniofacial sensory afferents, causing long-lasting hypersensitivity [8].

Corneal epithelium and perineural support cells (Schwann cells in peripheral facial nerves and satellite glial cells in the trigeminal ganglion) perform critical immunoregulatory functions. When injury disrupts the corneal epithelium or perineural tissue, the loss of this immunoregulation amplifies the effects of pro-inflammatory cytokines [8]. In the cornea, epithelial barrier dysfunction exposes subbasal nerve plexus terminals to mediators. In craniofacial regions, nerve injury disrupts the blood-nerve barrier, exposing axons to systemic and local inflammatory signals and perpetuating sensitization [9].

Hypersensitivity and persistently increased activation of corneal and craniofacial sensory afferents recruit dendritic cells, macrophages, and other immune cells to injured sites, accompanied by elevated levels of IL-1β, IL-6, IL-9, and TNF-α, which further enhance pain signaling and drive neuroinflammation [8]. This chronic neuroinflammation, combined with ectopic firing and altered ion channel expression in damaged nerves, sustains peripheral nociceptor hyperexcitability and triggers spontaneous pain, allodynia, and hyperalgesia in both ocular and facial domains. Persistent peripheral sensitization establishes the foundation for central sensitization and chronic neuropathic pain, even after apparent tissue healing [10].

### 2.2. Central Sensitization and Affective Amplification

The transition from peripheral to central sensitization in craniofacial and corneal neuropathic pain involves complex pathophysiology characterized by neuroinflammatory spread from peripheral terminals through the trigeminal ganglion to the spinal trigeminal nucleus (subnucleus caudalis) in the brainstem [8]. Chronic excitatory input from peripheral nerves is thought to produce several key changes though it should be noted that much of the supporting evidence derives from animal models and extrapolation from broader neuropathic pain the literature, with direct human validation in craniofacial and corneal populations remaining limited: (1) Hyperactivation of second-order neurons: second-order neurons in the trigeminal nucleus undergo enhanced synaptic transmission and expansion of receptive fields, constituting central sensitization [10]. Whether these changes are reversible with regenerative intervention in humans remains an open question. (2) Glial cell activation: microglia and astrocytes in the trigeminal nucleus and higher brain regions (thalamus, somatosensory cortex, and anterior cingulate cortex) are hypothesized to become reactive, releasing the same pro-inflammatory cytokines described in Section 2.1 and further amplifying pain signaling through synaptic modulation [8,11]. This mechanism is well-established in rodent models of trigeminal pain but has not been directly quantified in human corneal or craniofacial neuropathic pain cohorts. (3) Maladaptive neuroplasticity: evidence from both animal models and functional neuroimaging in broader chronic pain populations suggests that changes in gene expression, receptor density (including upregulation of NMDA and AMPA glutamate receptors), and synaptic strength may perpetuate central hyperexcitability [10]. Direct demonstration of these changes in human trigeminal pain conditions is currently lacking.

Under normal conditions, descending inhibitory pathways from the periaqueductal gray (PAG), rostral ventromedial medulla (RVM), and limbic system modulate ascending pain signals from the trigeminal ganglion via serotonergic, noradrenergic, and GABAergic mechanisms. It is hypothesized that in chronic craniofacial and corneal neuropathic pain, these inhibitory systems become dysfunctional through two primary mechanisms: (1) diminished GABAergic inhibition, whereby reduced GABA efficacy or loss of inhibitory interneurons in the trigeminal nucleus impairs pain modulation [8]; (2) a shift toward descending facilitation in which pathways that normally suppress nociceptive signals instead amplify them [12]. These mechanisms are supported by preclinical data and are inferred in human populations from therapeutic response patterns, but direct neurophysiological confirmation in craniofacial and corneal pain specifically remains an active area of investigation. Affective and cognitive factors such as anxiety, depression, and catastrophizing, are processed in limbic and prefrontal cortical circuits are established modulators of descending pain pathways in the broader chronic pain literature and are assumed to operate similarly in these conditions, contributing to affective amplification of pain perception [8,12].

Collectively, the features described above, particularly the prominence of central sensitization, altered descending modulation, and cognitive-affective amplification, confer nociplastic characteristics upon these conditions, even when the initiating event is a clear peripheral nerve injury. Clinicians and researchers should recognize this mechanistic overlap when selecting outcome measures and interpreting the effects of peripherally targeted regenerative therapies.

Because craniofacial pain syndromes are initiated and, in most cases, maintained by abnormal peripheral nociceptive traffic, restoration of peripheral nerve integrity offers a principled “bottom-up” strategy for de-escalating established central sensitization. Three mechanisms support this position. First, sustained ectopic discharge from injured primary afferents is a dominant driver of homo- and heterosynaptic facilitation at second-order neurons in the trigeminal subnucleus caudalis and medullary dorsal horn; regenerative interventions that re-establish orderly axon architecture and receptor expression, including recombinant human NGF (cenegermin), PRP- and AST-delivered growth-factor combinations, and MSC-derived exosomes, can reduce this tonic drive and withdraw the input that maintains wind-up and long-term potentiation at central synapses. Second, peripheral nerve injury activates satellite glial cells in the trigeminal ganglion and microglia and astrocytes in the spinal trigeminal nucleus, producing a self-reinforcing neuroimmune loop (IL-1β, TNF-α, CCL2, BDNF) that amplifies central excitability; by attenuating the upstream distress signals (ATP, CSF1, chemokines) that recruit this cascade, peripheral anti-inflammatory and neurotrophic therapies can normalize satellite-glia/neuron cross-talk and permit neuroimmune quiescence in both ganglion and dorsal horn [13]. Third, much of the synaptic plasticity underlying early central sensitization is experience-dependent and bidirectional: maladaptive potentiation can be depotentiated when peripheral drive is removed before transcriptional and structural remodeling render the central circuit autonomous, defining a therapeutic window in which timely peripheral repair is disproportionately effective. An important caveat follows. Once a syndrome becomes predominantly nociplastic, maintained independently of ongoing peripheral input, peripheral regenerative therapy alone is unlikely to reverse it, and combination with centrally acting pharmacotherapy or neuromodulation becomes necessary. This asymmetry underlies the mechanism-based stratification introduced in the following section: regenerative strategies are expected to have greater impact where peripheral drive still dominates, and progressively diminishing impact as the phenotype migrates toward nociplastic maintenance.

### 2.3. Sex Differences in Craniofacial and Corneal Neuropathic Pain

Sex is a significant biological variable in craniofacial and corneal pain conditions, yet it remains underexplored in the regenerative medicine literature. Epidemiological data consistently demonstrate that women are disproportionately affected by the conditions discussed in this review. Temporomandibular disorders affect women at approximately twice the rate of men, with female sex being among the strongest demographic predictors of TMD chronification and severity [3,13]. Migraine affects women at roughly three times the prevalence of men, with hormonal fluctuations across the menstrual cycle, pregnancy, and menopause representing well-recognized triggers and modulators of attack frequency and severity [14]. Dry eye disease and corneal neuropathic pain similarly demonstrate female predominance, with postmenopausal women constituting a particularly vulnerable population [2].

The mechanistic basis for these sex differences is multifactorial. Gonadal hormones, principally estrogen, progesterone, and testosterone, modulate nociceptive thresholds, neuroinflammatory responses, and central pain processing through both genomic and non-genomic pathways. Estrogen exerts complex, concentration-dependent effects on trigeminal nociception, and all three major estrogen receptor subtypes (ERα, ERβ, and GPER) are expressed within the trigeminal system and migraine-relevant central structures [15]. ERα is distributed throughout the brain including the supraoptic nucleus, paraventricular hypothalamic nucleus, and pontine nuclei, and is expressed in trigeminal ganglion neuronal nuclei and fibers, where it co-localizes with CGRP and the CGRP receptor. ERβ is expressed in trigeminal ganglion cytoplasm, and GPER in the pontine nuclei, spinal trigeminal tract, and ganglion cell membrane. Notably, females have significantly greater numbers of ERα- and ERβ-expressing cells in the trigeminal ganglion compared to males, providing a direct neurobiological substrate for sex differences in craniofacial pain processing [15].

Sex differences are also relevant to the regenerative therapies reviewed here. PRP composition varies with biological sex and hormonal status: platelet count, growth factor concentrations (including PDGF, TGF-β, and VEGF), and inflammatory mediator profiles differ between men and women and across hormonal states in women [16]. These differences may influence both the potency and the anti-inflammatory versus pro-regenerative balance of PRP preparations, yet current clinical protocols do not account for sex or hormonal status in PRP preparation or dosing. Similarly, MSC-derived secretome composition and immunomodulatory capacity have been reported to differ by donor sex in preclinical studies, with potential implications for cell-based and exosome-based therapies, though clinical data specifically addressing this in craniofacial or corneal applications are currently lacking.

Future clinical trials of regenerative therapies for craniofacial and corneal neuropathic pain should incorporate sex as a biological variable in study design, stratification, and analysis. Outcome reporting should be disaggregated by sex, and where feasible, hormonal status should be captured as a covariate. Addressing these gaps will be essential for developing precision regenerative medicine approaches that are equitable and optimally effective across patient populations.

## 3. Types of Regenerative Therapies

The regenerative therapies discussed below fall into three mechanistic classes that are useful to distinguish when aligning mechanism-of-action with pain phenotype. Anti-inflammatory and trophic biologics (autologous serum tears, platelet-rich plasma, amniotic membrane) deliver locally concentrated growth factors and cytokine-modulating proteins to dampen neurogenic inflammation and support epithelial and axonal healing. Cellular and paracrine therapies (mesenchymal stem cells and their extracellular vesicles) deliver a broader cargo that modulates both the local immune milieu and neuronal signaling. Neurotrophic peptides and growth-factor mimetics (cenegermin, BPC-157, α-MSH analogs) target identified receptor systems (TrkA, melanocortin receptors) to drive nerve regeneration. Table 1 summarizes these modalities and their evidence and limitations. Table 2 applies a qualitative evidence-tier framework (Tier 1 = regulatory-approved with Phase III RCT data; Tier 2 = multiple small-to-moderate RCTs; Tier 3 = case series or exploratory RCTs; Tier 4 = preclinical only) adapted from GRADE [17].

### 3.1. Ocular Surface Biologics

Ocular surface biologics, autologous serum tears (AST), PRP/plasma rich in growth factors (PRP/PRGF), and amniotic derivatives, provide neurotrophic support and modulate inflammation in corneal neuropathic pain and ocular surface disease with dry-eye and/or neuropathic features. For clarity, aqueous-deficient dry eye is a tear-film-mediated disorder of ocular surface homeostasis arising from reduced lacrimal output, which may or may not be accompanied by corneal nerve abnormalities. In contrast, neuropathic corneal/ocular surface pain is a somatosensory disorder arising from damage to or sensitization of corneal nerves, which may occur with or without coexisting tear-film deficiency, and the two entities can present together, sequentially, or independently. AST and PRP deliver neurotrophic factors (NGF, IGF-1, and BDNF) and anti-inflammatory mediators that support corneal nerve survival, axonal regeneration, and epithelial healing. Amniotic derivatives provide anti-fibrotic, anti-inflammatory, and neurotrophic effects that may reduce nociceptor sensitization and attenuate neuropathic pain signals [8,18].

AST studies demonstrate symptomatic improvement and enhanced healing in neuropathic dry-eye states, though trials remain small and heterogeneous. PRP/PRGF trials report reduced pain and improved corneal nerve metrics on confocal microscopy, but results depend heavily on preparation, activation, and dosing protocols. Amniotic derivatives show promise in preclinical and select clinical studies, though investigators have not yet established robust neuropathic pain endpoints. These biologic therapies range not only in reported outcomes but also in their methods of clinical delivery. AST is administered as topical drops or periocular formulations. PRP/PRGF delivery occurs periocularly or topically, with dosing ranging from daily to weekly. Practitioners apply amniotic products as drops or grafts [1]. Autologous products generally demonstrate good safety but lack standardization due to donor variability and processing differences. PRP/PRGF and amniotic derivatives require GMP-compliant production and regulatory adherence, which varies by jurisdiction and complicates cross-study comparisons [8,18].

Evidence for ocular surface biologics in this space is classified as Tier 2 for autologous serum tears and amniotic-membrane products, and Tier 2–3 for PRP/PRGF eye drops, reflecting multiple small-to-moderate RCTs alongside preparation heterogeneity that limits pooled analysis (Table 2).

### 3.2. Platelet-Rich Plasma

PRP involves delivering autologous PRP into targeted craniofacial or peri-neural regions to support nerve healing and modulate local inflammation [19,20]. This approach augments peripheral nerve repair with the expectation that improvements in peripheral nociception may translate into reduced central sensitization or altered pain processing [10]. PRP delivers concentrated growth factors (PDGF, TGF-β, VEGF, and IGF-1) and bioactive molecules, including serotonin, thereby promoting axonal regeneration, Schwann cell support, and remodeling of the perineural microenvironment [21,22]. Preclinical models demonstrate support for nerve regeneration after peripheral nerve injury, with the potential to dampen peripheral sensitization and modulate central nociceptive processing via trigeminocervical pathways [20,23]. PRP injections demonstrate generally good tolerance, with risks primarily related to the injection procedure (pain, swelling, and infection). Batch-to-batch variability remains a concern, requiring standardization of preparation protocols, platelet concentration targets, and activation methods.

Evidence for PRP eye drops sits at Tier 2–3 and for peri-neural PRP infiltration at Tier 3, reflecting case series and pilot RCTs with protocol heterogeneity that limits comparability (Table 2).

### 3.3. Mesenchymal Stem Cells

MSCs derived from dental pulp, bone marrow, adipose tissue, umbilical cord, and other sources offer neural crest-related regenerative potential and immunomodulatory capacity relevant to craniofacial and corneal neuropathic pain [24]. These cells modulate the local milieu and support nerve repair through paracrine activity and extracellular vesicle release [25]. MSC-based therapies represent a rapidly evolving frontier in regenerative medicine, with applications extending from tissue repair to neuropathic pain management. MSCs act primarily through paracrine signaling and extracellular vesicle release, delivering anti-inflammatory cytokines (IL-10, TGF-β, and PGE2) and neurotrophic factors (NGF, BDNF, GDNF, and VEGF) that modulate the local environment to favor nerve repair and reduce neuroinflammation [8,25]. Immunomodulatory effects include macrophage polarization from pro-inflammatory (M1) to anti-inflammatory (M2) phenotypes and T-cell suppression [26,27]. MSC-derived exosomes carry microRNAs and growth factors that influence nerve regeneration, representing a cell-free approach with advantages in manufacturing, storage, and safety. These mechanisms may attenuate peripheral sensitization and reduce afferent nociceptive drive to central pathways [8]. Standardized processing mitigates, but does not eliminate, immunogenicity and tumorigenicity concerns [24].

Clinical evidence for mesenchymal stem cells in craniofacial and corneal neuropathic pain is currently Tier 3–4 with early-phase human trials and a broader preclinical profile (Table 2).

### 3.4. Exosomes and Extracellular Vesicles

Exosomes and extracellular vesicles are cell-free biologics that carry proteins, lipids, and nucleic acids. Manufacturers can produce them at scale under GMP conditions. Researchers are exploring these scalable regenerative modalities derived from parent cells in ocular and neuropathic contexts [25,28]. Exosomes deliver bioactive cargo, including microRNAs (miR-21 and miR-146a), growth factors (NGF, BDNF, GDNF, and VEGF), and immunomodulatory proteins that modulate inflammation, promote axonal growth, and influence Schwann cell behavior. In ocular and trigeminal applications, exosomes provide neurotrophic support, anti-inflammatory effects, and regulation of angiogenesis and nerve repair, recapitulating many of the therapeutic effects of MSCs without the complexities of live cell transplantation [28,29,30]. Ophthalmic and trigeminal models show promising preclinical data. In corneal injury models, MSC-derived exosomes accelerate epithelial healing, increase corneal nerve density on IVCM, and reduce inflammatory cytokines (IL-1β and TNF-α) compared to controls [5,8,30]. Exosomes demonstrate the capacity to modulate corneal neovascularization and lymphangiogenesis while promoting nerve regeneration [28]. Early clinical data remain exploratory, with ongoing ophthalmology trials examining exosome applications for dry eye disease, corneal wound healing, and neuropathic pain [1,30]. Phase I/II trials report safety and preliminary efficacy signals, though CNP-specific outcomes as primary endpoints remain limited [29].

Allogeneic products offer off-the-shelf availability but raise immunogenicity concerns with repeated administration [31]. Key manufacturing challenges include standardized isolation methods, characterization requirements (particle size, surface markers, and cargo profiling), and validated potency assays correlating with clinical efficacy [29,30].

Evidence for MSC-derived extracellular vesicles is Tier 4, preclinical, with early Phase I safety data with no approved human product (Table 2).

### 3.5. Peptides and Neurotrophic Factors

This subsection covers targeted peptide therapies and neurotrophic-factor mimetics designed to promote nerve repair or modulate immune responses relevant to craniofacial and corneal neuropathic pain. Cenegermin is the most established example, with other peptides such as BPC-157 and alpha-MSH derivatives in various stages of investigation. Cenegermin (recombinant human nerve growth factor, rhNGF) directly supports corneal nerve regeneration and epithelial healing through TrkA receptor binding, activating downstream PI3K/Akt and MAPK/ERK signaling cascades that promote axonal growth, suppress apoptosis, and enhance epithelial proliferation, with downstream normalization of corneal sensation [18,32,33]. NGF also supports survival and differentiation of sensory and sympathetic neurons more broadly.

Cenegermin is the most established peptide therapy for corneal neurotrophic keratitis (CNK) and serves as a reference point for NGF-based corneal neuroregeneration [32,33]. In pivotal multicenter, randomized, vehicle-controlled Phase II and III trials, Pflugfelder et al. and Bonini et al. demonstrated that cenegermin promoted corneal epithelial healing (complete healing in ~70% of patients with moderate-to-severe NK) and supported nerve regeneration assessed by IVCM in patients with CNK, with favorable safety and tolerability profiles [32,33].

BPC-157 (body protection compound-157), a synthetic pentadecapeptide derived from gastric juice protein BPC, and alpha-MSH (alpha-melanocyte-stimulating hormone) derivatives are being explored for immune-guided repair and anti-inflammatory effects. Proposed mechanisms include dampening neuroinflammation, promoting angiogenesis, modulating cytokine profiles, and supporting tissue repair in various injury models [34]. However, mechanisms in craniofacial and corneal neuropathic pain remain incompletely characterized.

These results led to regulatory approvals by the FDA (2018) and EMA (2017) and establishing cenegermin as the standard against which investigators evaluate investigational peptides—such as BPC-157 and alpha-MSH derivatives—in CNK and related corneal neuropathic conditions [18,33]. Post-marketing surveillance and real-world evidence studies continue to refine the understanding of optimal patient selection and long-term outcomes. In contrast, BPC-157 and alpha-MSH derivatives remain largely preclinical or in early-phase clinical evaluation [34]. Preclinical studies suggest potential for nerve repair, anti-inflammatory effects, and tissue protection, but high-quality randomized controlled trials in trigeminal or corneal neuropathic pain are lacking. Alpha-MSH derivatives have shown immunomodulatory properties in ocular inflammation models but require validation in neuropathic pain contexts [34]. Cenegermin has received regulatory approvals (FDA 2018 and EMA 2017) and is associated with generally mild, transient adverse events (eye pain, hyperemia, and foreign body sensation), though its high cost, refrigeration requirements, and frequent dosing limit accessibility [33]. Other peptides (BPC-157, alpha-MSH derivatives) remain investigational without regulatory approval or established manufacturing standards [34]. The approval pathway of cenegermin therefore serves as a benchmark for future development of neurotrophic peptide therapies (see Section 5.1).

### 3.6. Safety Considerations

Safety profiles vary across the modalities reviewed and should be weighed against the severity of the pain syndrome treated. Blood- and amnion-derived biologics (autologous serum tears, PRP, amniotic products) carry predominantly local, self-limited adverse events. Cell-based therapies raise qualitatively different concerns: MSCs carry a theoretical risk of tumorigenicity, ectopic tissue formation, and, for allogeneic products, allo-immunogenicity, although clinical signals at current doses have been infrequent [35]. MSC-derived extracellular vesicles likely reduce these risks, but long-term human safety data remain limited, as summarized in Table 3.

## 4. Evidence in Clinical Application

Although corneal neuropathic pain, temporomandibular disorders, trigeminal neuralgia, and migraine present distinct clinical phenotypes, they share convergent pathophysiology within the trigeminocervical complex. Primary afferents from the ophthalmic (V1), maxillary (V2), and mandibular (V3) divisions, along with upper cervical C1–C2 afferents, all converge on second-order neurons in the trigeminal nucleus caudalis and the upper cervical dorsal horn [36]. Peripheral sensitization of these afferents, central sensitization in the trigeminocervical complex, and maladaptive descending facilitation thus represent a shared nociceptive architecture that each condition reviewed below engages to varying degrees. The discussion that follows therefore, treats these entities as related manifestations of trigeminocervical dysregulation rather than as unrelated clinical silos.

### 4.1. Corneal Neuropathic Pain

CNP is a particularly challenging entity in which patients experience persistent ocular surface pain symptoms—such as burning, photophobia, foreign body sensation, and irritation—in the absence of proportionate clinical signs of corneal pathology. This discordance between symptoms and clinical findings distinguishes CNP from other forms of dry eye disease and underscores the need for mechanism-based therapeutic approaches that address underlying nerve dysfunction rather than merely supplementing the tear film.

A terminological note is warranted here. The diagnostic label “corneal neuropathic pain” reflects the condition’s origin in peripheral corneal nerve damage and aligns with the IASP definition of neuropathic pain as pain caused by a lesion or disease of the somatosensory nervous system. However, as detailed in Section 2, the mechanistic profile of established CNP—particularly in patients with prominent discordance between symptoms and objective clinical findings—frequently includes features of nociplastic pain, including central sensitization, altered descending modulation, and cortical reorganization. The term “neuropathic” in the disease name and the term “neuropathic pain” as a mechanistic category therefore carry different meanings in this context. Clinicians should recognize that patients presenting with CNP may fall along a spectrum from predominantly peripheral (neuropathic) to predominantly central (nociplastic) pain mechanisms, and that this distinction has direct implications for therapeutic selection: peripherally targeted regenerative therapies such as cenegermin and autologous serum tears are most likely to benefit patients with active peripheral nerve pathology and low corneal nerve density on IVCM, while patients with preserved nerve density and prominent central sensitization features may require adjunctive centrally acting treatments.

The pathophysiology of CNP, as detailed in Section 2, involves peripheral sensitization of corneal nociceptors following initial insult (refractive surgery, infection, trauma, and autoimmune disease), followed by central sensitization when afferent pain signals become amplified and sustained at the level of the trigeminal nucleus and higher cortical centers [37]. Since conventional lubricants and anti-inflammatory therapies fail to address this underlying neural pathology, regenerative approaches targeting nerve repair and immunomodulation are possible therapeutic strategies [1,38].

Cenegermin is the most extensively studied and clinically validated regenerative therapy for CNP-related conditions. Cenegermin received regulatory approval for neurotrophic keratitis based on pivotal trials demonstrating corneal nerve regeneration, epithelial healing, and improved corneal sensitivity [33]. While the approved indication is neurotrophic keratitis rather than neuropathic pain per se, the mechanistic overlap—both conditions involve corneal nerve damage and dysfunction—has led to off-label exploration in CNP populations. Clinicians have treated patients with post-LASIK neuropathic pain and other forms of refractory ocular surface pain with cenegermin in case series, with variable but often favorable responses in symptom reduction and nerve density recovery as measured by IVCM. However, robust randomized trials specifically targeting CNP endpoints are lacking, and the high cost of cenegermin limits accessibility [32,33].

AST and PRP-PRGF are more accessible, though less standardized, regenerative options for CNP. AST contains neurotrophic factors such as NGF, IGF-1, and BDNF at physiological concentrations, along with anti-inflammatory mediators that may dampen peripheral nociceptor sensitization [18,39]. Clinical experience with AST in CNP is largely observational, with retrospective case series reporting symptomatic improvement in 50–70% of patients with refractory dry eye-like symptoms, particularly those with reduced corneal nerve density on IVCM.

Amniotic membrane products, available as cryopreserved grafts or lyophilized extracts for topical application, offer anti-inflammatory, anti-fibrotic, and neurotrophic properties that may benefit CNP patients. These products contain high levels of growth factors, extracellular matrix components, and anti-inflammatory cytokines. Clinicians have used amniotic membrane application in severe ocular surface disease and neurotrophic keratitis, with case reports suggesting symptom relief in CNP, though controlled trials with neuropathic pain-specific endpoints are absent. The mechanisms by which amniotic products might attenuate neuropathic pain include reducing surface inflammation (which can perpetuate nociceptor sensitization), promoting epithelial healing (which may restore epithelial-neuronal signaling), and providing direct neurotrophic support [40,41].

Nerve growth factor analogs beyond cenegermin, including investigational peptides such as BPC-157 and alpha-MSH derivatives, are in preclinical or early clinical development for CNP. BPC-157 has shown nerve-protective properties in animal models of peripheral nerve injury, though human data in ocular applications remain limited. Alpha-MSH derivatives, which act through melanocortin receptors to modulate immune responses, have demonstrated neuroprotective effects in preclinical models of ocular inflammation and may represent future adjunctive therapies for CNP [34].

Exosomes and extracellular vesicles are cell-free alternatives to MSC-based therapies for CNP. Exosomes derived from MSCs or corneal epithelial cells carry neurotrophic factors, microRNAs, and anti-inflammatory mediators that can modulate the corneal microenvironment and support nerve regeneration. Preclinical studies have demonstrated that topical or subconjunctival administration of MSC-derived exosomes promotes corneal nerve recovery and reduces inflammation in models of dry eye and nerve injury. Clinical translation is in early phases, with ongoing trials exploring exosome eye drops in dry eye disease; extension to CNP indications is anticipated as manufacturing and regulatory frameworks mature [29,42].

A critical advantage of regenerative therapies in CNP lies in the ability to monitor treatment response using IVCM, which quantifies corneal nerve density, nerve fiber length, and morphological features in real time. This imaging modality enables biomarker-driven patient selection (identifying patients with low nerve density who may benefit most from neurotrophic therapies) and objective assessment of treatment efficacy. Integration of IVCM metrics with patient-reported outcome measures (e.g., the Ocular Pain Assessment Survey and the Neuropathic Pain Symptom Inventory adapted for ocular use) can refine therapeutic decision-making and accelerate the attainment of clinical trial endpoints [18,37].

### 4.2. Temporomandibular and Myofascial Pain

Temporomandibular disorders (TMD) represent a range of conditions, including myofascial pain and temporomandibular joint (TMJ) osteoarthritis, that cause pain in the jaw, TMJ, and masticatory muscles. One in ten people experience symptoms severe enough to require treatment [13]. TMD pathophysiology involves biomechanical dysfunction, inflammatory joint changes, muscle hyperactivity, and, in many cases, peripheral and central sensitization of trigeminal nociceptive pathways [3,43]. Conventional TMD management follows a graded approach starting with conservative measures (patient education, behavioral modifications, and physical therapy), progressing to pharmacotherapy (NSAIDs, muscle relaxants, tricyclic antidepressants, and gabapentinoids), and advancing to interventional procedures, commonly targeting the temporomandibular joint directly, when conservative measures fail [43]. Interventional options have historically included intra-articular corticosteroid injections, hyaluronic acid viscosupplementation, botulinum toxin injections, and, in refractory cases, surgical interventions including arthrocentesis and arthroscopy [44,45].

Several studies support the efficacy of PRP in TMJ osteoarthritis [46,47,48]. Hegab et al. (2015) conducted a randomized trial comparing intra-articular PRP (1 mL injections administered weekly for 3–5 consecutive weeks) with hyaluronic acid in patients with TMJ osteoarthritis [49]. Results demonstrated significant pain reduction and improved mandibular movement with PRP compared to hyaluronic acid at 12-month follow-up while improvement peaked in the hyaluronic acid group and started to decline at 6-month follow-up.

A meta-analysis evaluating multiple intra-articular treatment options for TMD indicate that PRP ranks favorably among available treatments in terms of pain reduction and functional improvement [50]. Notably, corticosteroids—which may cause cartilage destruction when injected intra-articularly—are no longer recommended as first-line therapeutic injections for TMD, making PRP and hyaluronic acid the preferred options [3]. When researchers combined PRP with arthrocentesis (joint lavage), they found the combination superior to arthrocentesis alone in multiple studies [51]. However, when comparing PRP injection after arthrocentesis to hyaluronic acid injection after arthrocentesis, outcomes showed no significant differences [45]. Interestingly, studies have demonstrated that mixtures of hyaluronic acid and PRP prove superior to mixtures of hyaluronic acid and corticosteroids in pain relief, indicating potential synergistic effects of these treatment combinations.

Studies using platelet-rich fibrin (PRF), a second-generation platelet concentrate that releases cytokines via finer fibrin polymerization, have also shown favorable outcomes compared with hyaluronic acid for maximal mouth opening and pain reduction [52]. The proposed mechanism involves the sustained release of growth factors from the fibrin scaffold, potentially providing longer-lasting regenerative effects than those of liquid PRP formulations.

Recently, orthobiologic therapies, including PRP and cell-derived biologic products, have attracted growing interest for their potential regenerative and anti-inflammatory effects. A recent systematic review compared the outcomes of orthobiologic injections versus hyaluronic acid following TMJ arthrocentesis. Overall, orthobiologic therapies demonstrated potential benefits in reducing pain and improving maximum mouth opening (MMO). However, among studies directly comparing PRP with hyaluronic acid, most reported no statistically significant differences between the two treatments. In contrast, cell-derived orthobiologics showed significantly greater improvements in both pain reduction and MMO compared with hyaluronic acid. Collectively, these findings suggest that while orthobiologics—particularly cell-derived products—may offer promising therapeutic advantages in TMJ-OA management, the evidence remains inconsistent, and further well-designed clinical trials are required to confirm their clinical superiority [53].

Myofascial pain, which distinctly involves muscle-related pain with trigger points, commonly affects the masticatory system. Conventional treatment follows a graded system with conservative measures and medications (tricyclic antidepressants and selective norepinephrine-reuptake inhibitors), followed by interventions including dry and wet needling into trigger points [43]. Nitecka-Buchta et al. (2019) demonstrated that three intramuscular injections of 0.5 mL PRP into the masseter muscles bilaterally provided significantly greater pain relief compared to saline injections [54]. Agarwal et al. (2022) found that PRP injections combined with dry needling provided greater pain relief when compared to dry needling alone, suggesting additive effects of regenerative and mechanical interventions [55].

However, when Yilmaz et al. (2021) [56] compared dry needling with local anesthetic injection (mepivacaine 3%) to dry needling with PRP to botulinum toxin injection alone, botulinum toxin demonstrated the greatest improvement in pain at three months. This finding suggests that while PRP offers regenerative potential, established neuromodulatory agents may provide superior symptomatic relief in some myofascial pain phenotypes [56]. The mechanisms likely differ: botulinum toxin reduces muscle hyperactivity by inhibiting acetylcholine release, whereas PRP promotes tissue repair and modulates inflammation. Patient selection based on dominant pathophysiologic mechanism (muscle hyperactivity versus tissue injury/inflammation) may optimize treatment outcomes.

PRP’s therapeutic effects in TMD likely involve multiple mechanisms: delivery of growth factors (PDGF, TGF-β, and VEGF) that promote cartilage repair and synovial healing in TMJ osteoarthritis; anti-inflammatory effects through modulation of cytokine profiles; and in myofascial applications, promotion of muscle fiber repair and modulation of peripheral nociceptor sensitization [21]. The presence of serotonin in platelet dense granules may contribute additional neuromodulatory effects, though this mechanism requires further investigation in TMD contexts [57].

Since PRP represents an autologous, minimally manipulated product in most protocols, it typically falls under Section 361 HCT/P regulation in the United States, simplifying regulatory pathways. However, substantial variability in preparation protocols (platelet concentration targets, leukocyte content, activation methods, dilution for intra-articular use) creates significant challenges for cross-study comparisons and evidence synthesis.

### 4.3. Trigeminal Neuralgia and Post-Herpetic Neuralgia

Trigeminal neuralgia (TN) and post-herpetic neuralgia (PHN) are distinct but mechanistically related craniofacial neuropathic pain syndromes that may benefit from regenerative approaches targeting peripheral nerve repair and neuroimmune modulation [58]. The most common sites of treatment for trigeminal neuralgia include accessing the gasserian ganglion at the foramen ovale or at the peripheral branches (V1, V2, V3). Classical TN presents as episodic, lancinating facial pain that typically affects one or more divisions of the trigeminal nerve. Investigators often attribute classical TN to neurovascular compression at the trigeminal root entry zone, while secondary TN arises from structural lesions (e.g., multiple sclerosis plaques and tumors). Despite the predominance of neurovascular mechanisms, peripheral nerve damage and demyelination play critical roles in pain generation, making nerve-regenerative therapies a logical, though underexplored, adjunct to conventional treatments.

Current management of TN relies on first-line anticonvulsants (carbamazepine and oxcarbazepine) and second-line options (lamotrigine, gabapentin, and botulinum toxin type A), with surgical options (microvascular decompression, gamma knife radiosurgery, and percutaneous rhizotomy) reserved for refractory cases, consistent with current treatment guidelines. However, these interventions do not address underlying nerve injury or promote repair, and many patients experience recurrence or persistent dysesthesias following ablative procedures. Regenerative therapies may offer a complementary strategy by promoting Schwann cell remyelination, axonal repair, and modulation of neuroinflammatory cascades within the trigeminal ganglion and peripheral branches [23,59].

Researchers have explored PRP perineural injections in small case series for refractory TN, particularly in patients with secondary trigeminal neuropathy following trauma or iatrogenic injury. Perineural PRP administration near affected trigeminal branches (e.g., infraorbital and mental nerves) aims to deliver growth factors (PDGF, VEGF, and TGF-β) that support nerve regeneration and reduce ectopic discharge [60]. Zarembinski et al. proposed PRP as a novel treatment reported sustained pain relief in 5 of 8 patients with post-traumatic trigeminal neuropathy following ultrasound-guided PRP injections, with improvements maintained at 6-month follow-up. While these preliminary data encourage further investigation, controlled trials comparing PRP to conventional nerve blocks or sham injections are needed.

MSCs represent a more mechanistically targeted approach for TN, given their capacity to modulate neuroinflammation, support Schwann cell function, and promote axonal regeneration. Preclinical studies in rodent models of trigeminal nerve injury have demonstrated that perineural or systemic MSC administration reduces pain behaviors, decreases inflammatory cytokine expression in the trigeminal ganglion, and promotes nerve regeneration [31,61]. However, clinical translation for TN remains in early phases. A single case report described improvement in refractory TN following autologous adipose-derived MSC injection near the affected nerve, but investigators have published no formal clinical trials. The immunomodulatory effects of MSCs may prove particularly relevant in secondary TN associated with autoimmune conditions (e.g., multiple sclerosis), where neuroinflammation drives ongoing pain [26,27].

Exosome-based therapies offer a cell-free, scalable alternative to direct MSC administration. MSC-derived exosomes carry neurotrophic factors, anti-inflammatory microRNAs, and extracellular matrix-remodeling proteins that can modulate the microenvironment of the trigeminal ganglion and the peripheral nerve milieu. Preclinical data in peripheral nerve injury models suggest that exosomes promote Schwann cell proliferation, myelin repair, and reduction in neuropathic pain behaviors [29,30,62].

PHN, a complication of herpes zoster affecting trigeminal dermatomes (most commonly the ophthalmic division, V1), presents a distinct regenerative opportunity. PHN results from viral-mediated nerve damage, with persistent pain driven by peripheral nociceptor sensitization, dorsal root ganglion hyperexcitability, and central sensitization. Conventional therapies include gabapentinoids, tricyclic antidepressants, lidocaine patches, and capsaicin, with limited efficacy in many patients. Regenerative strategies for PHN aim to repair nerve damage and modulate the neuroinflammatory milieu that perpetuates pain. Topical cenegermin or NGF analogs could theoretically promote nerve repair in affected skin territories, though investigators have not tested this application in trials.

MSC-derived exosomes, delivered via perineural injection or even topical application in ophthalmic PHN, represent a novel approach supported by preclinical evidence of nerve repair and anti-inflammatory effects in herpetic models [32,33]. Practitioners have anecdotally used autologous serum or PRP-PRGF skin injections for PHN-related allodynia and dysesthesias, on the rationale that growth factors may normalize sensitized cutaneous nociceptors. A prospective, single-arm, open-label clinical study (n = 45) showed lower pain scores in patients with PHN after intradermal PRP injections into affected dermatomes [63].

### 4.4. Occipital Neuralgia and Migraine

Occipital neuralgia manifests as a chronic headache disorder characterized by paroxysmal or continuous pain in the distribution of the greater, lesser, or third occipital nerves. Trauma, compression, injury, or inflammation may trigger these nerves, which represent peripheral branches from cervical roots C1 and C2, leading to peripheral sensitization and ion channel upregulation [4]. The greater occipital nerve (GON) is the most common target for interventions in occipital neuralgia, as it sends afferent signals to the trigeminocervical complex (TCC). The TCC serves as a critical relay station for both cervical afferents and trigeminal nociceptors. Neurons in the nucleus caudalis, located from the medulla extending to the first cervical dorsal horns, converge at the TCC with the GON [64].

Initial treatment involves conservative management with NSAIDs, followed by muscle relaxants, gabapentin/pregabalin, and tricyclic antidepressants. When pharmacotherapy fails, interventional options include botulinum toxin injections, occipital nerve blocks (typically with local anesthetics and corticosteroids), radiofrequency ablation, occipital nerve stimulation, and surgical decompression [4].

Stone et al. (2024) conducted a double-blinded randomized trial comparing bilateral 2 mL injections of PRP to bilateral injections of 20 mg Depo-Medrol mixed with 1.5 mL of 2% lidocaine versus 2 mL of isotonic 0.9% saline [65]. Results demonstrated that PRP in occipital neuralgia provided pain relief comparable to saline and superior to steroid/anesthetic combinations at 3-month follow-up. This finding challenges conventional practice and suggests that PRP’s regenerative mechanisms may offer sustained benefit beyond transient local anesthetic or anti-inflammatory effects. The proposed mechanism involves PRP-mediated nerve repair and modulation of the perineural inflammatory milieu. By delivering concentrated growth factors to the GON region, PRP may promote Schwann cell function, reduce ectopic discharge, and dampen peripheral sensitization [19,20]. Through modulation of peripheral inputs to the TCC, PRP may indirectly influence central trigeminal pain processing, with implications for both occipital neuralgia and migrainous conditions involving trigeminocervical convergence [64].

While auras, allodynia, and other limbic system-related prodromal symptoms of migraines favor central nervous system involvement in migraine etiology, current therapies increasingly target peripheral nociception. The role of substance P, calcitonin gene-related peptide (CGRP), and other neuropeptides in the trigeminovascular system has long been established [66]. Notably, migraine medications targeting these nociceptors work outside the blood–brain barrier, supporting the therapeutic relevance of peripheral mechanisms [14]. Recently, researchers have highlighted platelets’ role in migraine pathophysiology through their involvement in nerve regeneration and synaptic plasticity, with effects dependent on IGF-1 and VEGF in motor function recovery [23,59]. Platelets may contribute to migraine pathophysiology not only through neuromodulation but also via the serotonin pathway, as platelets contain 90% of peripheral serotonin in dense granules [57]. Migraine patients experience relief from serotonin reuptake inhibitors and serotonin receptor antagonists, suggesting serotonin’s role in migraine pathogenesis.

Conventional migraine treatments range from first-line pharmaceutical agents like triptans (since 1991) to botulinum toxin injection treatments (since 2010). The known mechanisms of these treatments vary widely, reflecting migraine’s complex pathophysiology, but many share overlap in affecting platelet function, from NSAIDs to serotonin modulators. While researchers must conduct more studies to elucidate PRP’s effects on serotonin levels and metabolism, PRP holds potential to provide targeted therapy for migraine patients in analogous function to trigger point injections.

Trigeminocervical convergence and treatment implications: The significance of the TCC in occipital neuralgia and migraines makes the GON and other cervical trigger points attractive targets for PRP injections. By modulating peripheral inputs at the cervical level, clinicians may influence central trigeminal processing, potentially benefiting both occipital neuralgia and migraine phenotypes with prominent neck pain or occipital components [4,64].

## 5. Barriers to Translation

Translation of regenerative medicine approaches from preclinical promise to clinical practice faces multiple interconnected barriers spanning regulatory uncertainty, clinical evidence gaps, workforce readiness, and healthcare system integration.

### 5.1. Regulatory Uncertainty

For regenerative medicine approaches targeting craniofacial and corneal neuropathic pain, regulatory uncertainty—particularly regarding the concepts of minimal manipulation and homologous use—poses a major barrier to clinical translation. Under the human cells, tissues, and cellular and tissue-based products (HCT/P) framework, a product must satisfy all criteria in 21 CFR 1271.10(a) [67] to qualify as a Section 361 HCT/P, which faces substantially reduced regulatory burden. Failure to meet these criteria generally results in regulation as a drug, device, or biological product requiring premarket approval. FDA guidance indicates that, in the absence of definitive evidence, the agency will presume processing constitutes more than minimal manipulation and intended uses extending beyond a tissue’s basic function in the donor will be considered non-homologous.

Since regenerative strategies for neuropathic pain often depend on biologic mechanisms such as trophic support, anti-inflammatory signaling, or neural repair—functions that may not be recognized as basic donor functions—developers face significant uncertainty regarding regulatory classification. This uncertainty compounds because FDA guidance documents are nonbinding and reflect only the Agency’s “current thinking.” As a result, sponsors must often rely on case-by-case determinations through mechanisms such as the Tissue Reference Group or Requests for Designation. For neuropathic pain indications, where intended clinical effects often extend beyond the traditional structural roles of source tissues, ambiguity in applying homologous use standards can discourage investment and delay translation. FDA’s acknowledgment that regenerative medicine is complex and rapidly evolving, and that its regulatory interpretations may continue to change, further undermines the stability of regulatory expectations.

Regulatory frameworks vary substantially across jurisdictions. The European Medicines Agency (EMA) regulates most regenerative medicine products as Advanced Therapy Medicinal Products (ATMPs), requiring marketing authorization through centralized procedures. Japan’s Pharmaceuticals and Medical Devices Agency (PMDA) has implemented conditional approval pathways for regenerative medicines, allowing earlier market access with post-marketing surveillance requirements. These divergent regulatory approaches create challenges for multinational development programs and limit the portability of clinical evidence across regions.

Cenegermin was approved by the FDA as a biologic (BLA) in 2018 and by the EMA through the centralized procedure in 2017 under orphan designation for neurotrophic keratitis; Japan’s PMDA has not granted marketing authorization, and Japanese access is limited to clinical-trial or compassionate-use pathways. Autologous PRP is generally administered in the United States under the practice-of-medicine exemption for physician-prepared blood products, whereas in the European Union PRP preparations that are more than minimally manipulated are classified as Advanced Therapy Medicinal Products (ATMPs) requiring centralized authorization. Mesenchymal stem cell products illustrate a third regulatory pattern: the PMDA conditional/time-limited approval pathway established under the 2013 Pharmaceuticals and Medical Devices Act has permitted earlier market access to products such as Temcell for acute graft-versus-host disease, in contrast to the IND/BLA route required in the United States, where the FDA’s Regenerative Medicine Advanced Therapy (RMAT) designation can accelerate review but does not itself grant approval [68]. Table 4 summarizes regulatory status across these jurisdictions.

### 5.2. Clinical Evidence Gaps

The clinical success of regenerative therapies for neuropathic pain is constrained by deficiencies in evidence generation, outcome measurement, and patient selection strategies. Although expert consensus supports individualized, mechanism-based treatment approaches, most large-scale clinical trials do not incorporate predefined stratification [69]. Sensory phenotype stratification is the most feasible approach for large trials and is supported by evidence of distinct symptom clusters in conditions such as diabetic painful neuropathy. However, investigators rarely apply such stratification in regenerative medicine trials for craniofacial pain, likely due to the logistical complexity of incorporating quantitative sensory testing in early-phase studies with smaller sample sizes.

For corneal applications, IVCM provides objective biomarkers of nerve pathology that could guide patient selection—identifying patients with low nerve density who may benefit most from neurotrophic therapies versus those with preserved nerve density whose pain may reflect predominantly central mechanisms. Quantitative IVCM metrics with established reproducibility, such as corneal nerve fiber density (CNFD), corneal nerve branch density (CNBD), and corneal nerve fiber length (CNFL) can be utilized in future regenerative medicine trials, measured at a pre-specified device at baseline, 3 months, and 6 months.

Current trials employ heterogeneous outcome measures, complicating cross-study comparisons and meta-analyses. Many studies use generic pain scales (Visual Analog Scale and Numeric Rating Scale) without neuropathic pain-specific instruments. The field needs broader adoption of validated measures such as: Neuropathic Pain Symptom Inventory (NPSI), painDETECT questionnaire, Ocular Pain Assessment Survey (OPAS) for corneal applications, Patient-Reported Outcomes Measurement Information System (PROMIS) pain interference domains. At a minimum, future trials should report a neuropathic-pain-specific instrument (NPSI or NPSI-ocular for corneal applications), a condition-specific symptom instrument (OPAS for corneal applications; painDETECT or PROMIS pain interference for craniofacial applications), quantitative sensory testing at no fewer than two anatomically relevant sites, and durability outcomes at ≥6 months. Integration of patient-reported outcomes with objective measures (IVCM nerve metrics, quantitative sensory testing, and neurophysiologic assessments) would provide multidimensional outcome assessment and potentially reveal dissociations between structural repair and symptom improvement. Many published studies suffer from: small sample sizes with insufficient statistical power, short follow-up periods inadequate to assess durability, lack of appropriate control groups (sham procedures and standard-of-care comparators), heterogeneous treatment protocols precluding pooled analyses, and publication bias favoring positive results.

Current evidence for regenerative approaches to craniofacial and corneal neuropathic pain spans the spectrum from preclinical models to case reports to small, randomized trials. High-quality evidence from adequately powered, multicenter randomized controlled trials with validated endpoints remains sparse. This evidence gap limits regulatory approval pathways, clinical practice guideline development, insurance reimbursement policies, and clinician and patient confidence in treatment selection. Closing these gaps will require a formal Delphi consensus process, analogous to IMMPACT for chronic pain trials and TFOS DEWS II for ocular surface disease, to convert the domains identified above (imaging biomarker of sub-basal nerve architecture, neuropathic-pain phenotyping, condition-specific symptom burden, quantitative sensory testing, and durability) into a comprehensive outcome set for regenerative medicine trials in craniofacial and corneal neuropathic pain.

### 5.3. Workforce Development and Education Deficits

Translation of regenerative medicine into clinical practice is constrained by deficiencies in professional education and workforce development. Regenerative medicine is not currently a required component of medical education, and no standardized curriculum or formal board certification process exists [70]. This lack of structured training has prevented the development of a clearly defined workforce capable of safely and effectively delivering cell-based therapies. Educational gaps include limited exposure to regenerative medicine principles in medical school curricula, insufficient coverage of cellular therapies in residency and fellowship training, a lack of continuing medical education programs with standardized competencies, the absence of simulation-based training for complex injection techniques, and inadequate education on regulatory requirements and quality standards.

## 6. Conclusions

Craniofacial and corneal neuropathic pain impose substantial burdens on patients’ quality of life and frequently resist conventional therapeutic interventions. The complex pathophysiology—involving peripheral nociceptor sensitization, neuroinflammation, central sensitization, and maladaptive descending modulation—demands treatment approaches that address underlying structural and functional nerve pathology rather than merely suppressing symptoms. Regenerative medicine offers a paradigm shift from symptom palliation to tissue repair and functional restoration. Autologous biologics (serum tears and PRP), MSCs and their derivatives, exosomes and extracellular vesicles, and neurotrophic peptides target the root causes of neuropathic pain: damaged sensory nerves, dysregulated immune responses, and impaired nerve-support cell function.

Clinical evidence supporting regenerative approaches varies across modalities and indications. Cenegermin has achieved regulatory approval for neurotrophic keratitis, supported by robust Phase III trial data, though applications for corneal neuropathic pain remain off-label. PRP demonstrates promising results in TMJ disorders and myofascial pain, with growing evidence in occipital neuralgia. MSCs and their derivative exosomes show strong preclinical efficacy and early clinical promise, though large-scale human trials remain limited. Across all modalities, the field requires standardized protocols, validated neuropathic pain-specific outcome measures, and head-to-head comparative trials

Furthermore, sex as a biological variable remains critically underexplored across all regenerative modalities reviewed here. Given the well-established female predominance in temporomandibular disorders, migraine, dry eye disease, and corneal neuropathic pain, and the potential influence of gonadal hormones on both pain mechanisms and regenerative therapy composition, future clinical trials should incorporate sex-stratified design, analysis, and outcome reporting as a standard requirement.

This review has several limitations reflecting its scope and design. It is a narrative review; we did not apply a systematic review protocol, a PRISMA 2020 flow, or a formal risk-of-bias assessment of included studies. Literature selection, while guided by the SANRA framework (Section Methodology), relied on author judgment and is therefore subject to selection bias. The qualitative evidence-tier framework applied in Section 3 is adapted from GRADE but is not a formal GRADE adjudication. Conclusions drawn about emerging modalities, such as MSC-derived extracellular vesicles, are based on a rapidly evolving preclinical and early-clinical literature and may require substantive revision as Phase II and III trial data accumulate. We anticipate updating this framework as higher-tier evidence becomes available.

Three specific translational lessons from corneal research deserve emphasis as this field matures. First, in vivo confocal microscopy has established the feasibility and yield of objective nerve-structural biomarkers (corneal nerve fiber density, branch density, and length) for patient selection and outcome assessment; the underused trigeminal analogue, skin-biopsy intraepidermal nerve fiber density (IENFD), could deliver a similar structure-function linkage in craniofacial regenerative trials. Second, the cenegermin dose schedule (20 µg/mL topical, six times daily for eight weeks) is currently the only validated dose anchor for recombinant human NGF in any neuropathic pain indication and offers a defensible starting point for dose-ranging of NGF-pathway agonists in trigeminal neuropathic pain, subject to route-dependent adjustment. Third, corneal data increasingly indicate that peripherally targeted regenerative therapy is most effective when applied before central sensitization becomes self-sustaining, a pattern likely to generalize to trigeminal and craniofacial syndromes and reinforcing the case for early, mechanism-stratified patient selection.

As regenerative strategies mature from experimental interventions to evidence-based therapies, they could transform treatment for some of medicine’s most challenging pain conditions. Realizing this potential will require sustained investment in mechanistic research, rigorous clinical trials, multidisciplinary collaboration, and patient-centered outcome assessment. The unique advantages of the craniofacial and corneal domains—combining accessible anatomy, real-time imaging capabilities, and well-characterized nerve biology—position them as ideal platforms for developing and validating regenerative approaches that may ultimately benefit the broader population suffering from chronic neuropathic pain.

## Figures and Tables

**Table 1:** Overview of regenerative therapies for craniofacial and corneal neuropathic pain.

Therapy	Mechanism Class	Primary Indication	Current Clinical Stage	Key Limitations
Autologous serum tears (AST)	Anti-inflammatory/trophic	Neurotrophic keratitis; neuropathic corneal/ocular surface pain; severe dry eye	Widely used off-label; multiple small–moderate RCTs	Variable growth-factor content; non-standardized preparation; limited access/cold-chain
PRP/PRGF eye drops	Anti-inflammatory/trophic	Neurotrophic keratitis; corneal neuropathic pain; refractory dry eye	Small–moderate RCTs and comparative trials	Preparation-parameter heterogeneity (DEPA/PAW classifications inconsistently reported)
PRP peri-neural/ trigger-point infiltration	Anti-inflammatory/trophic	Post-surgical trigeminal and occipital neuralgia; TMD-associated neuropathic pain	Case series and pilot RCTs	Heterogeneous protocols; no Phase III data; operator variability
Amniotic membrane (cryopreserved/ lyophilized)	Structural-matrix/anti-inflammatory	Persistent epithelial defects; neurotrophic keratitis; neuropathic ocular surface pain	FDA-registered devices; small RCTs	Short retention; cost; donor-derived variability
Mesenchymal stem cells (dental pulp, BM, adipose, UC)	Paracrine/neurotrophic/immunomodulatory	Preclinical neuropathic pain; early-phase TMD and TN trials	Preclinical and early-phase clinical	Theoretical tumorigenicity; source and donor heterogeneity; cost; regulatory burden
MSC-derived exosomes/extracellular vesicles	Paracrine/neurotrophic	Preclinical corneal and trigeminal neuropathic pain models	Preclinical/Phase I	No potency assay standard; regulatory uncertainty; scale-up challenges
Cenegermin (recombinant human NGF, 20 µg/mL)	Neurotrophic (TrkA agonism)	Moderate-to-severe neurotrophic keratitis	FDA (2018) and EMA (2017) approved; Phase III RCT	High cost; transient ocular AEs; narrow indication; limited adult data outside NK
BPC-157 pentadecapeptide	Neurotrophic/anti-inflammatory (putative)	Preclinical nerve-injury models	Preclinical only	No approved human indication; regulatory and safety profile uncharacterized
α-MSH analogs (e.g., PL-9643, afamelanotide class)	Anti-inflammatory/melanocortin-mediated	Preclinical corneal nerve repair	Preclinical/early Phase	Systemic CV and pigmentary concerns with class; translation early

**Table 2:** Qualitative Evidence Tiers Across Regenerative Modalities.

Section	Modality	Evidence Tier	Best Evidence Available	Notes
3.1 Ocular Surface Biologics	Autologous serum tears	Tier 2	Multiple small-to-moderate RCTs + meta-analysis	Heterogeneous preparation across series
3.1 Ocular Surface Biologics	Amniotic membrane	Tier 2	Device-registered products; multiple small RCTs	Processing varies by manufacturer
3.2 Platelet-Rich Plasma	PRP/PRGF eye drops	Tier 2–3	Small-moderate RCTs; comparative studies	DEPA/PAW reporting inconsistent
3.2 Platelet-Rich Plasma	PRP peri-neural injection (craniofacial)	Tier 3	Case series and pilot RCTs	Protocol heterogeneity limits pooled analysis
3.3 Mesenchymal Stem Cells	MSC (various sources)	Tier 3–4	Early-phase human trials; preclinical corpus	Source/dose/route highly variable
3.4 Exosomes/Extracellular Vesicles	MSC-EVs	Tier 4	Preclinical + Phase I safety data	No approved human product
3.5 Peptides & Neurotrophic Factors	Cenegermin (rhNGF)	Tier 1	Phase III RCT; FDA (2018)/EMA (2017) approval	Only regulatory-approved neurotrophic therapy for neurotrophic keratitis
3.5 Peptides & Neurotrophic Factors	BPC-157	Tier 4	Preclinical animal models only	No human regulatory approval
3.5 Peptides & Neurotrophic Factors	α-MSH analogs	Tier 4	Preclinical and early clinical	Early translational stage

**Table 3:** Safety Comparison Across Regenerative Modalities.

Modality	Common AEs	Serious AEs	Theoretical Risks	Overall Risk
Autologous serum tears	Transient stinging or irritation	Rare microbial contamination	Blood-borne pathogen (autologous, low); contamination if preparation protocol breached	Low
PRP/PRGF eye drops	Transient irritation	Rare infection	Variable growth-factor content; processing contamination	Low
PRP peri-neural infiltration	Procedural pain; local swelling	Rare infection; neurovascular injury	Off-target neuronal injection; hematoma	Low–moderate
Amniotic membrane	Discomfort on placement; dislodgement	Rare infection	Donor-derived pathogen (screened)	Low
Mesenchymal stem cells	Injection-site reactions	Rare ectopic tissue formation; embolism with IV route	Tumorigenicity from in vivo differentiation; allogeneic immunogenicity	Moderate
MSC-derived EVs/ exosomes	Limited human data	None reported to date	Residual cellular debris; bioactive cargo variability	Low (preliminary)
Cenegermin (rhNGF)	Transient eye pain, hyperemia, foreign-body sensation, photophobia	Rare	Long-term corneal sensitivity effects under study	Low
BPC-157	Limited human data	Not characterized	Unknown mutagenic/oncogenic potential	Unknown (investigational)
α-MSH analogs	Limited human data	Not characterized	Pigmentary effects (class); systemic CV concerns with systemic dosing	Unknown (investigational)

**Table 4:** Regulatory Status Across FDA/EMA/PMDA by Modality.

Modality	FDA (US)	EMA (EU)	PMDA (Japan)	Regulatory Notes
Cenegermin (Oxervate^®^)	BLA approved 2018 (neurotrophic keratitis)	Marketing authorization 2017 (centralized)	Not approved; access via clinical trial/compassionate use	Orphan designation in US/EU
Autologous PRP (ocular drops)	Physician-prepared blood product; not a marketed drug	Member-state dependent; ATMP rules apply if more-than-minimally manipulated	Conditional regenerative-medicine pathway available	Practice-based; society-guidance rather than approval
Autologous serum tears	Physician-prepared blood product; compounding pharmacies	Member-state regulated blood-derived medicinal product	Hospital-compounded; not a marketed product	No label indication anywhere
PRP peri-neural infiltration (craniofacial)	Practice of medicine; not an FDA-approved drug	Member-state regulated; variable oversight	Regulated under medical-practice statutes	Off-label; no approved products
Amniotic membrane (cryopreserved/dehydrated)	Section 361 HCT/P if minimally manipulated, otherwise 510(k) device/351	ATMP or medical device depending on processing	Handled under regenerative-medicine pathway	Processing and claims drive pathway
MSC products (allogeneic/autologous cellular)	IND required; Section 351 HCT/P; RMAT designation possible	ATMP under Committee for Advanced Therapies (CAT)	Conditional approval under PMD Act 2013	Japan’s pathway is distinctive for early market access
MSC-derived EVs/exosomes	Investigational; IND required	Investigational; likely ATMP	Emerging regulatory consideration	No approved products globally
BPC-157	Not FDA-approved; WADA S0 prohibited class	Not authorized	Not authorized	Not approved for any human indication
α-MSH analogs	IND only (corneal indications); afamelanotide approved for EPP	Not approved for ocular/craniofacial pain	Not approved	Class includes approved systemic products for other indications

## Data Availability

No new data were created or analyzed in this study. Data sharing is not applicable.

## Abbreviations

AST	Autologous serum tears
ATMPs	Advanced Therapy Medicinal Products
BPC-157	Body protection compound-157
CNK	corneal neurotrophic keratitis
EMA	European Medicines Agency
CGRP	calcitonin gene-related peptide
GON	Greater occipital nerve
IVCM	In vivo confocal microscopy
MSCs	Mesenchymal stem cells
MSH	Melanocyte-stimulating hormone
NPSI	Neuropathic Pain Symptom Inventory
NGF	Nerve growth factor
OPAS	Ocular Pain Assessment Survey
PAG	Periaqueductal gray
PHN	Post-herpetic neuralgia
PMDA	Pharmaceuticals and Medical Devices Agency
PRF	Platelet-rich fibrin
PRGF	Plasma rich in growth factors
PROMIS	Patient-Reported Outcomes Measurement Information System
PRP	Platelet-rich plasma
rhNGF	Recombinant human nerve growth factor
RVM	Rostral ventromedial medulla
TCC	trigeminocervical complex
TMD	Temporomandibular disorders
TMJ	Temporomandibular joint
TN	Trigeminal neuralgia
